# Supplementary material for: Specialized neurons in the right habenula mediate response to aversive olfactory cues
Source: eLife. 2021 Dec 8;10:e72345. doi: 10.7554/eLife.72345 (PMC8691842; doi:10.7554/eLife.72345)
Supplement: Supplementary file 1. [file elife-72345-supp1.docx]

**Supplementary file 1**

| Transgenic line | Gateway | Primers |
| --- | --- | --- |
| Tg(Xla.Tubb2:QF2;he1.1:mCherry)^c663^ | P5’E:  Xla.Tubb2 | Forward: 5’-GGGGACAACTTTGTATAGAAAAGTTGTCTAGACCCTGTCTGTTCCTGA-3’  Reverse: 5’- GGGGACTGCTTTTTTGTACAAACTTGAATCAGTGGATGGTGTGGCCCT-3’ |
|  | pME:  QF2-pA | Forward: 5’ -GGGGACAAGTTTGTACAAAAAAGCAGGCTCAACATGCCACCCAAGCG -3’  Reverse: 5’ - GGGGACCACTTTGTACAAGAAAGCTGGGTAAAAAACCTCCCACACCTCC -3’ |
|  | P3’E:  SV40pA;HE1.1:mCherry | Forward: 5’ - GGGGACAGCTTTCTTGTACAAAGTGGGATCCAGACATGATAAGATACAT -3’  Reverse: 5’ - GGGGACAACTTTGTATAATAAAGTTGCCATAGAGCCCACCGCAT -3’ |
| Tg(QUAS:mApple-CAAX; he1.1:mCherry)^c636^ | P5’E:  QUAS | Forward:  GGGGACAACTTTGTATAGAAAAGTTGGGGTAATCGCTTATCCTCGGATA  Reverse:  GGGGACTGCTTTTTTGTACAAACTTGACGCGTCTTCGAGGTCGAGGGA |
|  | pME:  mApple-CAAX | Tol2kit v2.0 (#768) from Kristen Kwan |
|  | P3’E:  SV40pA;HE1.1:mCherry | Same as above |
| Tg(QUAS:GCaMP6f)^c587^ | P5’E:  QUAS | Same as above |
|  | pME:  GCaMP6f | Forward: 5’-GGGGACAAGTTTGTACAAAAAAGCAGGCTACGCCGCCACCATGGGTTCTCATCATCATCATCATCAT- 3’  Reverse: 5’-GGGGACCACTTTGTACAAGAAAGCTGGGTTCACTTCGCTGTCATCATTTGTAC-3’ |
|  | P3’E:  polyA | Tol2kit v1.2 (#302) from Chi-Bin Chien |
| Tg(QUAS:loxP-mCherry-loxP-GFP-CAAX)^c679^ | P5’E:  QUAS | Same as above |
|  | pME:  loxP-mCherry-stop-loxP | Tol2kit v2.0 (#759) from Kristen Kwan |
|  | P3’E:  GFP-CAAX-pA | Forward: 5’ -GGGGACAGCTTTCTTGTACAAAGTGGAAATGGTGAGCAAGGGCGAG-3’  Reverse: 5’- GGGGACAACTTTGTATAATAAAGTTGAAAAAACCTCCCACACCTCCCCC-3’ |
| Tg(QUAS:loxP-mCherry-loxP-BoTxBLC-GFP)^c674^ | P5’E:  QUAS | Same as above |
|  | pME:  loxP-mCherry-stop-loxP | Tol2kit v2.0 (#759) from Kristen Kwan |
|  | P3’E:  BoTxBLC-GFP-stop | Forward: 5’ -GGGGACAGCTTTCTTGTACAAAGTGGAAATGCCCGTGACAATTAACAATT-3’  Reverse: 5’- GGGGACAACTTTGTATAATAAAGTTGTCACTTGTACAGCTCGTCCA-3’ |
